# O012. Intra-variability of the characteristics of migraine attacks

**DOI:** 10.1186/1129-2377-16-S1-A70

**Published:** 2015-09-28

**Authors:** Michele Viana, Grazia Sances, Natascia Ghiotto, Elena Guaschino, Marta Allena, Giuseppe Nappi, Peter J Goadsby, Cristina Tassorelli

**Affiliations:** Headache Science Centre, C. Mondino National Neurological Institute, Pavia, Italy; Headache Group, NIHR-Wellcome Trust Clinical Research Facility, King's College, London, London, UK; Department of Brain and Behavioral Sciences, University of Pavia, Pavia, Italy

## Background

Migraine attacks may have different features with respect to different patients and also within the same patient. The percentage of patients reporting attacks as stereotyped and those reporting attacks with different phenotypes has not been the object of specific investigations. Here we wanted to evaluate the percentage of migraine patients reporting attacks with the same characteristics, in terms of phenotype and response to symptomatic medications, on three consecutive attacks.

## Methods

Thirty patients with migraine without aura prospectively recorded the features of three consecutive attacks in a headache diary. Characteristics recorded were: pain intensity, presence of nausea, vomiting, photophobia, phonophophia, osmophobia, allodynia, cranial autonomic symptoms (at least one), and premonitory symptoms. Patients were allowed to take frovatriptan as symptomatic medication, whose efficacy was evaluated at the two hours pain-free rate.

## Results

None of the patients presented identical characteristics on the three studied attacks (Table 1). This was still the case if we reduced the number of variables evaluated from eleven to seven of the eight core features indicated by the ICHD. Considering just six variables: unilaterality, quality of pain, presence/absence of nausea, vomiting, photophobia and phonophobia, only two patients (6%) had identical features on three consecutive attacks.

**Table 1:** Migraine attack features and intrapatient variability.

Unilateral location	Pulsating quality	Pain intensity (4 point scale)	N	V	PT	PN	O	A	CAS	PS	Number (%) of patients with three identical attacks with respect to some of the features recorded at the moment of symptomatic medication intake
**x**	**x**	**x**	**x**	**x**	**x**	**x**	**x**	**x**	**x**	**x**	**0 (0%)**
**x**	**x**	**x**	**x**	**x**	**x**	**x**					**0 (0%)**
**x**	**x**		**x**	**x**	**x**	**x**					**2 (6%)**
			**x**	**x**	**x**	**x**					**9 (30%)**
**x**	**x**										**10 (33%)**

N: nausea, V: vomiting, PT: photophobia, PN: phonophobia; O: osmophobia; A: allodynia; CAS: cranial autonomic symptoms (at least one); PS: premonitory symptoms (at least one).

With respect to the response to frovatriptan, 39% of patients had the same outcome (positive or negative pain-free in 2 hours) on three consecutive attacks.

## Conclusions

To the best of our knowledge this is the first study that systematically assessed the percentage of patients reporting migraine attacks with identical features in a given period. Our results demonstrate that migraine attacks show a high variability not just among patients, but also within the same patient. Our findings indicate that stereotype of attacks is uncommon, and reinforces the underlying logic of the current operational classification system.

Written informed consent to publication was obtained from the patient(s).

## Conflict of interest

None.

